# A Role for the Intestinal Microbiota and Virome in Myalgic Encephalomyelitis/Chronic Fatigue Syndrome (ME/CFS)?

**DOI:** 10.3390/jcm5060055

**Published:** 2016-06-06

**Authors:** Navena Navaneetharaja, Verity Griffiths, Tom Wileman, Simon R. Carding

**Affiliations:** 1 The Gut Health and Food Safety Research Programme, The Institute of Food Research, University of East Anglia, Norwich NR4 7UA, UK; navena.navaneetharaja@ifr.ac.uk (N.N.); t.wileman@uea.ac.uk (T.W.); 2 Norwich Medical School, University of East Anglia, Norwich NR4 7TJ, UK; v.griffiths@uea.ac.uk

**Keywords:** myalgic encephalomyelitis, chronic fatigue syndrome, intestinal microbiota, dysbiosis, microbiome, virome

## Abstract

Myalgic encephalomyelitis/chronic fatigue syndrome (ME/CFS) is a heterogeneous disorder of significant societal impact that is proposed to involve both host and environmentally derived aetiologies that may be autoimmune in nature. Immune-related symptoms of at least moderate severity persisting for prolonged periods of time are common in ME/CFS patients and B cell depletion therapy is of significant therapeutic benefit. The origin of these symptoms and whether it is infectious or inflammatory in nature is not clear, with seeking evidence of acute or chronic virus infections contributing to the induction of autoimmune processes in ME/CFS being an area of recent interest. This article provides a comprehensive review of the current evidence supporting an infectious aetiology for ME/CFS leading us to propose the novel concept that the intestinal microbiota and in particular members of the virome are a source of the “infectious” trigger of the disease. Such an approach has the potential to identify disease biomarkers and influence therapeutics, providing much-needed approaches in preventing and managing a disease desperately in need of confronting.

## 1. Introduction

### What Is ME/CFS?

Descriptions of outbreaks of illness resembling those of myalgic encephalomyelitis/chronic fatigue syndrome (ME/CFS) have been reported for more than two hundred years with the term benign myalgic encephalomyelitis proposed in the late 1950s as a name for a group of symptoms including protracted muscle pain with paresis, emotional disturbances and encephalitis [1]. As a result of an outbreak of illness in Lake Tahoe, USA in the mid-1980s, the name CFS describing the main symptom of the illness was proposed, with the first case definition for CFS being produced in 1988 [2]. Diagnosis does however remain difficult, relying principally on exclusion, with the current estimate of 0.1%–0.2% of the population being affected [3] and multiple studies showing that it can take more than five years for patients to be diagnosed [4]. Affected individuals are predominantly women and minority groups, of an average age of 33, though the disease has been reported to affect those as young as 10 and as old as 77 [5,6,7,8]. At least one-quarter of patients are house- or bed-bound at some point in their lives [5,9] with as few as 6% of individuals returning to pre-morbid levels of function [3]. The societal impact of ME/CFS is therefore considerable with the annual direct and indirect economic cost estimated to be between $17 and $24 billion [10].

The diagnosis of ME/CFS is commonly based on the Fukuda [11], Oxford [12] and/or the International Consensus Criteria (ICC) classifications [13], that identify the core symptoms of the condition as the presence of neuropsychological features such as a distinguishing post-exertional malaise, sleep disturbance and autonomic symptoms. A key distinction between classifications is the acknowledgment of persistent fatigue for six months or more, a requirement not made by the ICC classification. The Fukuda and Oxford classifications are not considered as ME-specific as the Canadian definition, which identifies symptom clusters of ME with emphasis on neurosensory, immune, gastrointestinal and genitourinary manifestations of the disease. While such discrepancies in diagnostic consensus are apparent, there is no doubt that their presence can lead to profound losses of daily function in a subset of patients. In a report published in 2015 The Institute of Medicine (The National Academies, Washington, D.C., USA) proposed a new diagnostic algorithm focusing on its central symptoms, emphasising that studies aimed at assessing the natural history of the disease and its temporal characteristics are essential for a better understanding of the causes, improved diagnosis and treatment of ME/CFS [14].

All ME/CFS case definitions include some inflammatory symptoms and signs although their prevalence and severity ranges widely across different studies that vary in time period, location and case definition. However, immune-related symptoms of at least moderate severity that persist for prolonged periods of time (>6 months) are commonly reported in ME/CFS patients [15]. The origin of these symptoms and whether it is infectious or inflammatory in nature is not clear with several studies seeking evidence of acute or chronic infections and/or underlying immune impairment in ME/CFS, which is discussed in more detail below. The recent link between ME/CFS and xenotropic murine leukemia virus-related virus (XMRV) [16] has largely been disproven and attributed to laboratory contaminants [17]. This highlights the need for further carefully controlled studies that use sensitive methodologies to detect infectious agents, that can produce findings which can be independently replicated in different cohorts of ME/CFS patients [18]. Here, we present a critique of the current knowledge and understanding of infectious agents in the pathogenesis of ME/CSF, with the aim of providing focus for further research, exploring the potential impact of the intestinal microbiome and virome using considered and sensitive study designs.

## 2. Infections and ME/CFS

Reports dating back more than 60 years describing disease outbreaks in “closed populations” such as in hospitals and convents producing symptoms consistent with ME/CFS raised the possibility of an infectious aetiology or, an association of infection with disease onset [1,19,20]. ME/CFS patients often present with flu like symptoms with an acute onset presentation being more common in these patients than in those suffering from chronic fatigue only [21]. Population based studies however, have shown a predominance of a gradual over acute infectious onset to be more consistent with a chronic infection or reactivation of a latent infection [8]. In view of this and the overlapping symptoms of ME/CFS with chronic virus infections, it is not surprising that numerous attempts have been made to identify viruses as a trigger of ME/CFS (Table 1 and Table 2).

### 2.1. Viral Infection

Numerous viruses have been associated with the initiation or perpetuation of ME/CFS, including Epstein Barr Virus (EBV) [22,23], Ross River virus [22], human herpesvirus-6 (HHV-6) [28], parvovirus B19 [29,32], hepatitis C virus, human retroviruses and enteroviruses [33]. Amongst these, EBV is most frequently identified as a possible viral trigger of ME/CFS (Table 1). However, in considering the difficulties of detecting active, pathological infections and distinguishing an active infection from reactivation of a latent infection, the vast majority of studies rely on indirect and/or anecdotal evidence. In particular, heavy reliance is placed on detecting the presence, or elevated levels, of virus-specific antibodies in ME/CFS patients’ sera. Whilst high titers of EBV antibodies that includes reactivity to viral capsid antigen (VCA) have been found in subsets of patients compared to healthy individuals who were previously infected with EBV [24,34], not all studies have replicated these findings [29,35,36,37,38,39,40].

In addition, the presence of serum antibodies to EBV, HHV-6 and CMV viruses are not specific to ME/CFS and are associated with other diseases and they can be found in asymptomatic individuals [41]. Also, elevated levels of these antibodies may result from unrelated conditions in subjects with an altered immune system that leads to virus reactivation. Other approaches that have been used to attempt to demonstrate causality include the use of animal models in which animals, usually mice, are inoculated with specific viruses or their products in an attempt to reproduce disease symptoms and through the use of antiviral medications in patients.

Glaser and colleagues used mice to investigate the contribution of EBV proteins expressed during viral replication in disease induction [42,43,44]. Stimulation of the mouse immune system by dUTPase can induce immune-modulatory effects that resemble the clinical symptoms observed in ME/CFS sufferers including reduction in body mass and physical activity that correlated with increased production of inflammatory cytokines (TNFα, IL-1β, IL-6) together with enhanced Natural Killer (NK) cell activity and IFNγ synthesis. Of note, EBV dUTPase was shown to stimulate production of cytokines by human PBMCs [45]. Although these studies suggest that viral products, such as non-structural EBV-encoded protein, can attach to cellular membranes, enter multiple organs and induce immune-modulatory effects that resemble the clinical symptoms observed in ME/CFS sufferers [46], such findings need to be corroborated using larger cohorts of patients and appropriate control groups.

The successful use of antiviral medication in ME/CFS patients could provide support for a virus infection as a trigger for ME/CFS or rather, a perpetuating factor for the disease. Montoya *et al.* conducted a double-blind, placebo controlled trial using the anti-viral agent valganciclovir in a total of 30 patients with elevated IgG antibody titers against either EBV or HHV-6. After 6 months, the treatment arm showed significant improvement in measurements of fatigue using the fatigue severity scale, along with significant beneficial changes in monocyte and cytokine levels [47,48]. However, the small numbers in each treatment arm limit the study findings, which are further exacerbated by reported high dropout rates by the end of the study. Furthermore, although high IgG titres are a surrogate marker for intense infection with both EBV and HHV-6, it may also be a sign of an affective immune system able to produce large amounts of antibody in response to infection.

A prospective study of patients with elevated serum antibody titers to EBV in addition to CMV or HHV-6 resulted in a 75% response rate to long-term valacyclovir and/or valganciclovir treatment, reporting significantly increased long-term Energy Index Point Scores, with improvements in cardiac, immunologic and neurocognitive abnormalities [49]. However, another double blind, placebo controlled trial of acyclovir in a small number of ME/CFS patients (*N* = 27) showed no clinical difference in treated *versus* placebo controls [50]. An alternate approach is based upon enhancing antiviral defences in ME/CFS patients in which it is assumed they have an unidentified, underlying viral infection and using various immune activating agents to boost the immune system and antiviral responses. A pilot single blind, placebo-controlled study measuring the clinical effect of the immunostimulatory drug Isoprinosine in sixteen CFS patients, resulted in the improvement of symptoms in 60% of patients. Patients who reported improved clinical parameters also showed significantly enhanced NK cell activity which correlated with the duration of therapy and to significantly increased numbers of CD4^+^ T helper cells and IL-12 levels at week 28 [51]. A recent clinical trial of Rintatolimod, an agonist of Toll-Like Receptor (TLR)-3 which, via recognition of double stranded RNA elicits production of anti-viral type 1 interferon, showed objective improvements in exercise tolerance and a reduction in symptoms compared to placebo after 40 weeks, indicating a microbial role in ME/CFS [52].

Evidence to the contrary that virus infection is an aetiological factor in ME/CFS has come from the analysis of monozygotic twins discordant for disease in which no differences in the virus profile and expression was found in healthy *versus* disease twin pairs [36]. Other studies comparing ME/CFS patients with healthy control subjects have failed to identify any significant differences in virus content and/or serum levels of antibodies specific for parvovirus B19 [53], HHV-6A, HHV-6B or HHV-7 [54,55,56,57]. The reasons for these apparent discrepancies are unclear but may be related to the inherent difficultly of detecting active infections and discriminating it from reactivation of latent infection, other unrelated conditions and immune alterations. They may also be related to the use of different disease classifications and patient inclusion/exclusion criteria and stratification and the use of different analytical methodologies. Many studies that rely on blood samples for detecting evidence of virus infection may miss enteroviruses, parvovirus, or herpes virus infections that continue to persist in the gut, brain or heart tissue, respectively.

A role for enteroviruses in ME/CFS has been suspected for many years [30,58,59] with the study of Chia and colleagues identifying a higher prevalence of enterovirus infection in gastric biopsy samples of ME/CFS patients with significant gastrointestinal symptoms (82%), compared to control subjects (20%) [31]. Others however, have found no increased incidence of enterovirus infection in ME/CFS patients [60] (Table 2).

### 2.2. Non-Viral Infection

The evidence of a bacterial infection as a trigger of ME/CFS has only been described in case reports of Q-fever and *Chlamydia pneumonia* and unlike studies focusing on viral infection, none have focused on the effect of antibacterial therapy on symptom relief in ME/CFS cohorts [61,62,63]. The association between ME/CFS and parasitic [64], or fungal [65] aetiologies have also been limited and inconclusive. Currently, there is insufficient evidence to conclude that ME/CFS is caused by a bacterial infection or that the disease is sustained by an ongoing infection.

## 3. Immune Impairment in ME/CFS

Studies of the immune status of ME/CFS patients are aimed at biomarker discovery and identifying distinguishing features of immune profile or function that can provide insights into the (immuno)pathogenesis of ME/CFS. The most consistent finding is a reduction in the function of NK cells that play an important role in immune surveillance and viral immunity [66]. Impaired NK cell function is a common finding in patients [67,68,69] although the absolute number of NK cells is not affected by disease states [39,67,68,69,70,71,72,73,74]. Overall, studies have lacked consistency and reproducibility, with different studies focusing on different identity markers and different indicators of cytotoxic activity. The current literature does not therefore convincingly establish a correlation between NK cell function and disease severity.

The usefulness of low NK cell cytotoxicity as a biomarker for ME/CFS is further questioned by a the presence of a similar phenomenon and impaired NK cell function in rheumatoid arthritis, cancer and endometriosis [75,76,77] as well as in healthy individuals who are older, smoke, psychologically stressed, physically deconditioned or sleep deprived [78,79,80]. Evidence of specific changes in NK function including altered perforin and granzyme concentrations [81] have also been described although these findings have not been independently verified.

Other indicators of immune alteration in ME/CFS patients come from the comparative analysis of cytokine levels in the blood or more rarely, other sites such as cerebrospinal fluid. The majority of studies are limited by examining a select few cytokines in small numbers of patients most of which have failed to yield significant findings. Despite this, some studies have reported raised levels of TNFα in ME/CFS patients [70,82,83,84] which is of particular interest as it is a mediator of malaise and a feature of CNS inflammatory disorders [85,86] and via its action on vaso-vagal reflex circuits in the brainstem can disrupt autonomic control [87]. However, the significance of such changes in TNFα or any other cytokine in ME/CFS patients is uncertain since they may simply reflect immune activation and can be altered in various chronic inflammatory disorders and are therefore unlikely to be specific to ME/CFS. Consistent with this interpretation are the results from a study using monozygotic twins discordant for ME/CFS that found no evidence of differences in pro-inflammatory cytokine levels in the affected *versus* non-affected twin [88]. The use of different sampling methods, diagnostic and inclusion criteria and sources of patients and control subjects may account for contrasting outcomes from immune function studies in ME/CFS patients, with some studies lacking suitably matched controls. Moreover, relatively few studies were longitudinal [68], often relying on a single sample and time point of analysis unable to account for the natural history of ME/CFS [6].

### 3.1. Is ME/CFS an Autoimmune Disease?

The positive outcome of clinical trials of Rituximab (trade names Rituxan, MabThera and Zytux), a chimeric monoclonal antibody that recognises the pan-B cell surface antigen CD20, in Norwegian cohorts of ME/CFS patients arguably provide the strongest evidence to date of immune alterations and dysfunction in ME/CFS [89] and that the disease may be autoimmune in nature [90] (see below). The drug has previously been successfully used in the treatment of rheumatoid arthritis [91]. Rituximab treated ME/CFS patients reported an overall response rate of 67% compared to 13% in the placebo arm, with significant reductions in fatigue scores after 8 months. The transient symptom improvement is consistent with a mode of action of Rituximab in depleting autoantibody-producing B cells and with an autoimmune-based pathogenesis of ME/CFS. Confirming these findings and positive Rituximab treatment outcomes in additional larger, multi-centre based trials would make the case for ME/CFS being an autoimmune disorder more compelling. Other immunophenotyping-based studies of ME/CFS patients that have described alterations in B cell populations [92,93] and greater numbers of naïve B cells, provide additional indirect evidence in support of this interpretation [94,95].

Identifying the origin and nature of the autoantigens that drive autoimmune responses are important in establishing ME/CFS as an autoimmune disorder. Several putative self-reactive antigens have been identified including serotonin, dopamine and cholinergic neurotransmitters [96,97] (Table 3). For example, one study has reported that individuals with ME/CFS have increased 5-hydroxytryptamine (HT) autoimmune activity associated with activation of inflammatory pathways and increased bacterial translocation which has led to analogies with neuroimmune aspects of multiple sclerosis [98]. How and where in the body autoreactive immune cells are activated is a matter of conjecture. However, given its continuous exposure to a vast number of microbes, immune cells and immune processes, we propose the gut as an attractive and important source of autoreactivity with the potential to cause chronic debilitating disease.

### 3.2. A Gut Origin for Autoimmunity?

A novel premise involving an autoimmune association in ME/CFS is of a microbial trigger introduced not by an externally acquired pathogen and infection, but by microbes already residing within that host (microbiome), the vast majority of which comprise the intestinal microbiota. Whilst alterations in the composition of the intestinal microbiota (dysbiosis) have been described and associated with different disease states in both experimental animals and humans, the most compelling causal evidence of the direct involvement and a requirement for the intestinal microbiota in disease initiation comes from the use of sterile, germ-free animal (mouse) models of autoimmune disease [106]. In most but not all of the disease models, that includes inflammatory bowel disease, autoimmune arthritis, Type 1 diabetes and systemic lupus erythematosus, the severity and/or incidence of disease is reduced or absent under germ-free conditions consistent with the microbiota being a ‘trigger’ for disease progression. It is important to note however, that the identity of the pathobionts able to promote disease after colonization (conventionalisation) of the germ-free mice has not been established for any of these diseases. The absence of a mouse model that faithfully reproduces the hallmark features of human ME/CFS is a constraint and limiting factor in determining if or how the microbiota contributes to the pathogenesis of ME/CFS.

How do members of the intestinal microbiota gain exposure to the hosts’ immune system? Intestinal dysbiosis is thought to promote or be associated with altered permeability of the intestinal epithelial barrier, termed ‘leaky gut syndrome’. Disruption of epithelial junctional complexes that normally tightly bind adjoining cells (tight junctions) in the boundary epithelium provides a portal of entry for the translocation of bacteria or bacterial components into mesenteric lymph nodes or the systemic circulation [107,108]. This can, if the disruption is extensive and not effectively resealed lead to local and systemic immune cell activation and resulting production of pro-inflammatory mediators and cytokines that can further disrupt epithelial tight junction [109]. A pertinent example is the aetiology of Guillain-Barre syndrome, where *Campylobacter jejuni* lipopolysaccharide (LPS) that is structurally similar to host cell gangliosides leads to the production of cross-reactive antibodies that target and damage host neurones leading to paralysis [110].

The junctional complexes of intestinal epithelial tight junctions also display striking similarities to endothelial tight junctions in the blood-brain barrier (BBB), as seen in a study involving HIV-1 infected mice treated with LPS [111]. Systemic administration of LPS was shown to disrupt the integrity of the BBB, permitting the migration of HIV-1 infected monocytes to enter the brain from the peripheral circulation. LPS is thought to disrupt the intramembranous junctional complex proteins such as ZO-1 and occludin, compromising the structural integrity of the BBB. Activation of microglia can occur by LPS via its binding to CD14 and TLR-4, which initiates an inflammatory reaction within the cranial circumventricular organs [112]. Experimental studies have shown that chronic intestinal inflammation elicited by bacterial products can result in neurological disease, with bacteria-derived LPS thought to play a role in the generation of antibodies cross reactive with host lipoproteins and the aetiology of multiple sclerosis [113]. Of relevance to ME/CFS, one study has reported significantly higher titres of serum antibodies to LPS in ME/CFS patients with abdominal discomfort than in controls, concluding that gastrointestinal symptoms were associated with increased bacterial translocation, which is a potential driver of systemic inflammatory processes [82]. Support for this hypothesis has been obtained in independent studies reporting a correlation between the translocation of enteric bacteria with disease activity in subsets of ME/CFS patients [114]. The intestinal microbiota may therefore serve a role in initiating or perpetuating immune activation, bacterial translocation and autoimmune processes, which have all been implicated in the pathogenesis of ME/CFS [90].

## 4. Could the Intestinal Microbiota Play a Role in the Development of ME/CFS?

The co-existence of ME/CFS and gastrointestinal symptoms is well documented [115] with one study reporting that 92% of ME/CFS patients have co-existent irritable bowel syndrome (IBS) [116] and with studies of the immunological relationship between the two conditions showing increased mucosal and systemic levels of pro-inflammatory cytokines IL-6, IL-8, IL-1β and TNFα [117,118].

Initial analyses of bacterial populations using stool samples from ME/CFS patients found higher distributions of *Escherichia coli* as a percentage of total aerobic microbes compared to healthy controls (92.3% *versus* 49%), however these findings were reported in a scientific meeting, with unspecified methodology [119]. d-lactic acid producing *Enterococcus* and *Streptococcus* species were also found to be significantly increased in ME/CFS patients, which was confirmed by culture with NMR-based metabolic profiles showing that samples from ME/CFS patients were associated with lower levels of *Bifidobacteria* [120]. Other studies have extended these observations, showing an association between increasing *Enterococcal* counts and the severity of reported neurological and cognitive deficits [121], with a larger study describing an association between significant reductions in *Bifidobacteria*, higher aerobe to anaerobe ratios and more severe gastrointestinal functional deficits [122]. Furthermore, 77% of CFS patients have been found to have small intestinal bacterial overgrowth [123], which displays some similar clinical symptoms to ME/CFS [121].

To date, only one study has utilised sensitive next-generation sequencing technology to profile the microbiota in ME/CFS by comparing Belgian and Norwegian patients to healthy controls [124]. Discriminant function analysis revealed distinct microbiota populations in patients and controls in both countries based on bacterial genera (*p* = 0.022), with Norwegian patients displaying highly significant differences in intestinal microbiota compositions compared to Norwegian controls (*p* < 0.001), typified by significant variations in *Firmicutes* populations, with a 50-fold decrease (*p* = 0.00001) in the *Firmicutes* genera Holdemania and a 20-fold increase (*p* = 0.003) in Lactonifactor. The geographical differences in microbiota compositions were attributed to Norwegian patients reporting a longer duration of illness, suggesting that the intestinal composition is altered in ME/CFS patients. Differences in experimental protocols and bacterial DNA extraction techniques may explain the contrasting outcomes of these studies, and emphasizes the need to compare larger cohorts with reliable matched subjects. Several factors govern why microbiota profiling studies in ME/CFS lack apparent consistency including, patient related issues such as inconsistent phenotypic classifications, small and often unpowered sample numbers and technical issues related to differences in the reliability, sensitivity and coverage of microbial sequence detection provided by different DNA extraction and 16S RNA amplification protocols, different sequencing platforms and the reliance on single samples and time points of analysis that can only provide a snapshot of microbiota compositions. Despite demonstrations of altered diversity and stability of the intestinal microbiota in ME/CFS, it is not yet possible to say that a specific microbial signature exists in ME/CFS. Care must also be taken in attempting to establish such a disease microbiota signature in view of the heterogeneity in symptoms and subgroups presented in ME/CFS.

## 5. What Next for ME/CFS Microbiome Studies?

Microbiome studies have to date almost exclusively focused on defining changes in intestinal bacterial population; recently however, the remit has broadened to assess the non-bacterial microorganisms of the intestine. Of particular interest are the viral components of the microbiota. Components of the virome, specifically bacteriophages, which make up 90% of the gut virome composition [125] are primary drivers of bacterial diversity and influence community structure by both eliminating and introducing traits to their host species via the horizontal transfer of genes [126,127,128]. So far, the impact of the intestinal virome in healthy and disease states has received very little attention, but in its unexplored functional role it is possible that the phages in the virome will have as yet unexplored, explored effects. These can be indirect effects resulting from changes to bacterial populations, or direct effects following stimulation of the immune system if they cross gut epithelial barriers and enter the host.

### 5.1. The Intestinal Virome

The virome has been shown to be more personalized and stable than bacterial intestinal communities [129], with the profiling of viral communities in female monozygotic twins and their mothers finding limited intra-personal variation compared to high inter-personal viral variation [130]. This variability has been attributed to both the presence of individual intestinal bacteria and the rapidly evolving nature of viral populations. The longitudinal sampling of a healthy male over a period of 2.5 years revealed that 80% of viral contigs, which are contiguous reads of sequenced DNA, persisted over that period. Variations over time were attributed to diversity-generating retroelements, a family of genetic elements that serve to diversify DNA sequences and CRISPR arrays, short segments of prokaryotic DNA within bacterial and archaeal genomes that can establish if a microbial population has been previously infected by phage [130].

Virome sequencing has lagged behind bacterial microbiome studies; principally as a result of technical limitations [131]. Recent viral metagenomic studies have displayed limited capacities to assign viral sequences to taxa, with current databases lacking appropriate depth to identify viruses, let alone link specific bacteriophages to individual bacteria or disease states. Shotgun metagenomics is a high throughput technique that allows direct analysis of random pieces of DNA sequences from the genomes of whole samples and initial reads have been used to assemble complete genomes of phages and viruses that infect humans [132,133]. However, metagenomic sequencing analyses has often ignored RNA viruses, whose role in the intestinal microbiome remain unclear and current isolation procedures which are primarily designed to isolate DNA viruses, may have overlooked the presence of RNA viruses in the intestinal virome [134]. Prior attempts at characterizing the virome have only been able to assign 15% to 87% of virus-like particle sequences or contigs within sequence databases [129,130,135]. This variability is a limitation of current taxonomic assignment criteria and we are only able to report on bacteriophages that are most closely related to taxa in current databases. This is thought to be due to rapid evolution of phages and their sequences, although it can also be due to the initial quality of sequence reads and the proportion that were trimmed prior to analysis. It is clear that each individual displays different viral sequence diversity, of which the full representation of the resident viruses can only be obtained with deeper sequencing approaches. Such techniques are required to enable the identification of regions of viral diversity that associate with the virulence of specific viruses or disease. This is a process that requires the compilation of complete reference databases for gene families in addition to reads that are long enough to be identifiable.

With increasing interest and expanding evidence of the role of phages in governing disease states and the potential use of phages in combatting disease [136], interest has incorporated the role of viruses in normal human physiology, a prime area of research of which is the contribution of the intestinal microbiota in influencing the brain and behaviour as part of the gut-microbiota-brain axis.

### 5.2. The Gut-Microbiota-Brain Axis

A potential pathophysiological mechanism for ME/CFS development can be explained by the integrated microbiota-gut-brain-axis [137], which describes the physiological links between the microbiota and the central nervous system (CNS), the autonomic and enteric nervous system and the hypothalamic-pituitary-adrenal (HPA) axis. Increasing evidence indicates that the microbiota can, by as yet incompletely defined mechanisms and molecular mediators, communicate with the CNS via immune, neural and endocrine pathways, which are likely to have effects on cognitive function and behaviour [138].

Microbial interactions with the enteric nervous system have been demonstrated extensively using germ-free mice, which allows the impact of colonising bacteria (coventionalisation) on mood and cognitive function to be tested [139,140]. The physiological mechanisms by which stress can alter the microbiota are thought to be due to its effects on intestinal physiology, altering gastrointestinal motility and secretions via reductions in the waves of electrical activity (migrating motor complexes) that sweep through the intestine in a regular cycle and trigger peristalsis, increasing intestinal permeability and reducing the regenerative capacity of the intestinal mucosa, all of which serve to alter the natural environment of intestinal microbiota. Different microbial niches within the intestinal environment initiate the release of stress hormones such as norepinephrine, which can influence inter-bacterial signalling, the growth of specific pathogens in addition to their ability to adhere to the intestinal mucosa [141]. Sudo *et al.* were the first to demonstrate differences in brain function between germ-free and specific pathogen free (SPF) mice, with germ-free mice displaying exaggerated hypothalamic-pituitary responses to mild restraint stress, through increased levels of adrenocorticotrophic hormone (ACTH) and corticosterone release compared to SPF mice [142]. This was subsequently reversed upon conventionalisation in germ-free mice using faecal samples from the SPF mice. Sudo *et al.* also demonstrated increased stress responses in germ-free mice colonised with enteropathogenic *E. coli*, with subsequent reversal of stress-induced ACTH and corticosterone levels when germ-free mice were colonised with the human commensal *B. infantis*; the effect of which was partially reversed when germ-free mice were colonized with intestinal microbes from SPF mice. Interestingly, Sudo *et al.* displayed this reversibility in very young mice, indicating a potential stage in which neurones that regulate stress responses are sensitive to stimulation from the microbiota. Stress induced in early life has been demonstrated to lead to dysbiosis in germ-free mice and has been deemed to be a critical determinant of abnormal behaviours [143]. Furthermore, Heijtz *et al.* demonstrated the influence of the intestinal microbiota in influencing synaptic brain activity, with germ-free mice displaying increased motor activity and reduced anxiety in comparison with SPF mice with normal microbiota, indicating the influence of intestinal microbes in brain activity and psychiatric comorbidity [140].

Further studies have demonstrated the impact of host microbiota in controlling the maturation and function of microglia in the CNS [144]. Most significantly, Bercik *et al.* conducted the first study to demonstrate the ability for behavioral traits to be transferred between mouse strains that displayed anxious and aggressive behaviors strains using fecal microbiota transplantation (FMT) [145].

The study of associations between autistic spectrum disorder (ASD) and the intestinal microbiota has provided avenues for exploration in ME/CFS cohorts. Patients with ASD, like ME/CFS, have a high prevalence of gastrointestinal dysfunction [146], which has also been linked to alterations in microbiota composition [147,148]. A study exploring the connection between the microbiota and brain function in ASD showed patients to have marked alterations in the microbiota, with specific alterations in various *Clostridium* species [149]. Hsiao and colleagues showed a gut-microbiota-brain connection in an adolescent mouse model of ASD (maternal immune activation, MIA), which exhibits intestinal barrier defects and microbiota alteration [150]. Upon treatment of MIA offspring with *B. fragillis*, intestinal permeability was corrected, microbial composition was altered and ASD-related deficits in communication and behaviour were reduced. MIA offspring also displayed an altered serum metabolomics profile, specifically key molecules in a tryptophan metabolism pathway, including increased levels of serotonin that were restored to normal upon treatment with *B. fragillis* [151]. This work suggests the role of probiotic bacteria and bacterial metabolites in preventing or causing neurodevelopment disorders respectively, suggesting an important role for the microbiota in the development of social behaviour in ASD. Of possible interest in ME/CFS research is the sleep-inducing substance derived from bacterial cell walls, Factor-S. Studies have suggested that intestinal bacteria are an important source of Factor-S, especially after Brown demonstrated normal sleep patterns to be disrupted after perturbation of the microbiota with oral antibiotics [152].

Stress, or an organism’s ability to cope with environmental demands is well known to increase susceptibility to diseases that include gastrointestinal disorders [153]. It is also well established that dietary and environmental stresses in turn, create different microbial niches affecting the localisation of the different microbiota populations which can particularly favour pathogenic bacterial species [154]. This can lead to the release of stress hormones and cytokines such as IL-6 [142,155]. Bacteria too, can respond to and synthesize hormones and neurotransmitters with for example, *Lactobacillus* and *Bifidobacteria* species producing acetylcholine and/or gamma-amino butyrate (GABA); *Escherichia* species producing norepinephrine, serotonin and dopamine; and serotonin produced by *Streptococcus* and *Enterococcus* species [156]; all of which can contribute to the host’s regulation of mood, cognition, pain and anxiety.

There is clearly still much to be learned about the molecular connections and pathways involved in gut-microbiota-brain signalling and how perturbations of this communication pathway might give rise to disease states. The effect of the intestinal microbiota on the CNS and cognitive function represents exciting and potentially fruitful opportunities for future investigation and explaining the manifestation of the core symptoms of ME/CFS. Also, the advent of ‘biotics’ (probiotics and/or prebiotics) and FMT provides opportunities to manipulate the microbiota to improve or restore gut health and impact on how it influences the function of other organ systems, including the immune system and the CNS.

## 6. A Proposal for Future Research: Intestinal Dysbiosis Drives Autoimmunity in ME/CFS

It is likely that ME/CFS follows a non-classical autoimmune mechanism and it has been long described to encompass idiopathic immune dysregulation. Based upon the evidence presented in this review, a candidate for chronic stimulation of the immune system that triggers autoimmune processes may be found in the intestinal virome. It is worth stressing that should phylogenetic differences in the virome and microbiome be detected in ME/CFS patients, future research needs to be directed towards determining the consequences of these changes and their impact on the functionality of the microbiota. This could then provide insight into the adaptive mechanisms of commensals and potentially pathogenic microorganisms that are required for persistence in the GI-tract, in addition to describing how the microbiota shapes the development and function of the immune system in these patients.

The virome evidently plays a powerful part within the microbiome, with bacteriophages being shown to have a major impact on the dynamics and evolutionary processes of their host populations and the identification of novel viruses is of clear interest. Roux *et al.* recently showed microbial hosts to be specifically associated with particular viruses, suggesting a long-term co-evolution between the viruses and their host [157] and indicating that profiling bacterial communities will strengthen any association between virome composition and ME/CFS. As Norman *et al.* displayed in their study of the enteric virome in inflammatory bowel disease, the power of detecting disease-associated changes in the virome were only observed by comparing patients and household controls, controlling for the influence of genetics and environment [158]. Future studies will need to account for within-host variability in species level analysis by age and gender matching control participants.

The composition of the human gut virome shows significant variation to be dependent on bacterial populations and highlights the benefits of concurrent viral and bacterial community profiling in future microbiome studies. We propose that phages shape the bacterial microbiota through density-dependent predation, a mechanism by which actively replicating or reactivating phages lyse their bacterial hosts, change the abundance of specific bacterial species and induce dysbiosis [159]. Once lysed bacteria release their cell contents comprising of proteins and nucleic acids, these products serve as antigens and microbe-associated molecular patterns (MAMPS) that trigger mucosal host responses, for example, via MyD88-independent pro-inflammatory cytokine production displayed in *in vitro* models [160]. The ability of viruses to stimulate mucosal responses has also been considered by the constant re-infection of viruses of low virulence and humans being chronic carriers of viruses, which has been previously shown to be associated with increased risk of developing diseases such as asthma [161] and type 1 diabetes [162,163].

Viral-induced inflammation can lead to increased intestinal epithelial permeability resulting in the translocation of phages across mucosal surfaces and increased systemic exposure to microbial epitopes [127]. Oral administration of phages in animal studies have demonstrated translocation of phages to systemic tissues [164] and experimental studies of phage interactions with the immune system have found free phage particles to spread systemically via breaks in the intestinal mucosa or by dendritic cell transport, with phages being immunogenic enough to generate antibodies in human infants [165]. These studies suggest that mammals have mechanisms for the uptake and delivery of phage that allow intestinal phages to provoke innate and adaptive immune responses. For these reasons, phages may serve as innate immune ligands that stimulate host immunity and inflammation, characteristic of an environment of dysbiosis and autoimmunity.

Previous culture-based assays characterizing the bacterial components of ME/CFS patients have reported a reduction in the proportional abundance of *Bifidobacteria* species [120] that can contribute to maintaining intestinal epithelial integrity [166]. A reduction in these beneficial Gram-positive bacteria may favour the expansion and interaction of harmful Gram-negative bacteria within the intestinal mucosal surface, or by removing the beneficial traits of *Bifidobacteria* species thereby precipitating the symptoms of ME/CFS. For example, increased levels of *Bifidobacteria* have been associated with increased plasma tryptophan levels and serotonin and dopamine production in areas of the brain associated with depression and anxiety [167]. Additionally, studies have shown certain *Bifidobacteria* species to have a significant impact on functional improvements in NK cell activity and to lower levels of IL-4, IL-5 and IL-10 cytokines [168,169]. These effects support the presence of Th1 directed cell-mediated immunity [170,171], important for the control of viruses and bacteria. NK cells also interact with dendritic cells, which are in close contact with the gut epithelium and mount tolerogenic responses to preserve immune homeostasis in the gut [172]. Any disruption to this fine-tuned interface between bacterial species, immune function and epithelial cell barrier could give rise to inappropriate immune responses. Given the suggested immunomodulatory properties of *Bifidobacterium*, it is possible that the phages associated with *Bifidobacteria* species can via their influence on diversity and function of their host bacteria initiate such downstream immunological effects. Further studies are required investigating the interactions between keystone bacterial species, phages and the mucosal immune responses that may provide a more complete picture of the intestinal environment in ME/CFS.

As highlighted by Goodrich *et al.* identifying genes associated with members of the microbiota can provide valuable information on host-microbiota interactions [173]. Focus should now be on understanding how phage genomes relate to each other and their hosts, and the evolutionary mechanisms that shape phage populations. *In vivo* studies and animal models will strengthen metagenomics findings, providing insights into how resident phages and eukaryotic viruses shape commensal bacteria and their impact on host immunity and gene expression. A ‘bacteriophage adherence to mucus’ model has been used to show that concentrated phage bound to the intestinal mucus layer result in a dramatic decrease in bacterial attachment to the mucus and significantly decreased damage to cultured cells [174]. Deeper, more detailed sequencing approaches and expanding databases will certainly improve virus identification, accounting for the uncharacterized ‘dark matter’ of the human enteric virome.

## 7. Concluding Remarks

While studies have to date failed to identify a distinct microbial signature that establishes a pathogenic role of the intestinal microbiota in ME/CFS, a cycle of chronic intestinal dysfunction and instability of the microbiota certainly characterizes a subset of patients with ME/CFS. Pursuing the role of intestinal microbial dysbiosis in the pathophysiology of ME/CFS may well establish how immunological dysregulation manifests in its core symptoms. The use of next-generation sequencing techniques and metagenomic tools may identify predictors of disease relapse and chronicity [175], with the technology continually being tailored for use on viral ecosystems [176,177]. Analogous to IBD studies, microbiota characterization may elucidate more distinct subpopulations within current ME/CFS classifications. Ultimately, observing any association between microbial phylogeny and the ME/CFS disease phenotype can highlight the impact of the microbial community on human health, and has the potential to identify disease biomarkers and influence therapeutics, providing much-needed approaches in preventing and managing a disease in need of confronting.

## Figures and Tables

**Table 1:** Summary of studies of EBV infection and ME/CFS.

Study Participants (Diagnostic Criteria)	Study Design	Analysis	Findings	Reference
253 infected patients including 68 EBV+ (Fukuda); 16–77 years, 57% male; ≥3/12 SOMA scale for disease severity. Controls: Self-reported group	Prospective cohort following EBV infection	Serum Abs	28/253 (11%) developed ME/CFS following EBV	Hickie *et al.*, 2006 [22]
250 primary care patients with an URTI or glandular fever (Fukuda + Oxford); 16–65 years of age.	Prospective cohort	Interview and EBV serum Ab	47% acquired acute CFS after infection *cf.* to 20% after URTI (RR 2.3, 95% CI 1.3–4.1). CFS prevalence at 6 months post glandular fever was 9%–22% *cf.* 0%–6% after URTI (RR 2.7–5.1).	White *et al.*, 1998 [23]
2 cohorts of 63 and 387 patients (Fukuda); 27–63 and 20–78 years of age; 73% and 63% women respectively. Controls: 61 healthy controls	Comparing patients *vs.* controls	Serum EBV Ab and Ag	Attenuated T and B cell responses in CFS patients. EBNA-IgG titers reduced in 10% of CFS patients. Reduced number EBNA-1- and VCA-antibody secreting memory B cells in 76% of patients. EBV-induced secretion of TNFα and IFN-γ was significantly lower. Lower % EBNA-1-specific TNF-α/IFN-γ/IL-2+ CD4 and CD8 T-cells. EBV load in blood immune cells, EBER-DNA > BZLF-1 RNA in CFS patients suggesting more frequent latent replication.	Loebel *et al.*, 2014 [24]
58 (Fukuda + CDC); mean 44 years of age; 75% female. Controls: 68 matched controls; average age 53 years and 73% women	Case control	Serum anti-VCA and EA Ab	100% VCA and EA IgM+ when EBV positive. Diagnostic use in a ME/CFS subset?	Lerner *et al.*, 2004 [24]
14 (Sharp + Holmes), mean 35 years of age, 64% women	Cohort of travelers with CFS symptoms	Serum Abs	3/14 EBV IgM+ (11/14 CMV IgM+)	Gascon *et al.*, 1995 [25]
301 IM patients including 39 CFS patients (Fukuda); mean 16 years of age; 90% female; baseline characteristics predicted severity of CFS. Controls: 50 IM patients fully recovered by 6 months; age 16 years and 74% female)	Prospective nested case control longitudinal study	Self-reporting CFS	39 developed ME/CFS with 50 controls reporting full recovery.	Jason *et al.*, 2014 [26]
301 IM patients with 39 CFS patients (Fukuda); 12–18 years of age; 90% female; disease defined by length of time affected.	Cohort study determining ME/CFS incidence at 6, 12 and 24 mouths	Self-reporting ME/CFS	13% at 6 mouths 7% at 12 mouths 4% at 24 mouths	Katz *et al.*, 2009 [27]

Ab, antibody; RR, relative risk; CI, confidence interval; IM, infectious mononucleosis; UTRI, upper respiratory tract infection.

**Table 2:** Studies of non-EBV virus infections and ME/CFS.

Virus	Study Participants (Diagnostic Criteria)	Study Design	Analysis	Findings	References
Human Herpes Virus 6 (HHV-6)	35 ‘adults’ (CDC + Fukuda); 71% female; 27/35 with severe disease *vs.* 21 Multiple Sclerosis patients *vs.* 25 healthy controls	Case control	Serum anti-virus Abs	32/35 (91%) patients HHV-6 gp110a IgG+ *cf.* 88% *vs.* 22/25 controls. 23/35 (65.7%) patients HHV-6 p41/38a IgG+ *cf.* 20% *vs.* 5/25 controls.	Ablashi *et al.*, 2000 [28]
Parvovirus B19	48 (Fukuda); 37 ± 13 years of age; 78% female; severity not assessed *vs.* 35 otherwise healthy controls with mild GI upset; 66% female, 46 ± 17 years of age	Case control quantifying viral loads	PCR detection of virus in GI-biopsy and blood	38%–40% gastric and duodenum biopsies B19+ *cf.* 14% controls (OR 3.93, *p* = 0.008). No virus detectable in the blood.	Fremont *et al.*, 2009 [29]
Enterovirus	48 (Oxford); 35.3–37.5 mean age, 54% male, severity not assessed *vs.* 29 controls with ‘normal muscle’	Case control detecting virus RNA	RT-PCR detection in muscle biopsies	20.8% of biopsies virus RNA+ *cf.* none detected in controls. 9/10 enterovirus RNA+ patients had abnormal lactate response to exercise.	Lane *et al.*, 2003 [30]
	165 (CDC); ages, sex and disease severity not reported; duration of illness: 5.0 ± 4.5 years *vs.* 22 healthy controls *vs.* 12 gastric diseases	Case control study	RT-PCR detection viral RNA in GI-biopsy	82% of biopsies VP1+ *cf.* 20% of controls. ~33% of patients virus RNA+ over 4 years, 5 with transient growth of non-cytopathic enteroviruses.	Chia *et al.*, 2008 [31]

**Table 3:** Autoantibodies in ME/CFS patients.

Antigen Target of Autoantibodies	Reference
Cardiolipin	Hokama *et al.*, 2008 [99] Hokama *et al.*, 2009 [100]
Nuclear envelope antigens	Konstantinov *et al.*, 1996 [101]
Neuronal cell	Buchwald *et al.*, 1991 [102]
68/48 kD protein antibodies	Nishikai, M., 2007 [103]
Serotonin, microtubule-associated protein 2 and muscarinic cholinergic receptor-1	Bassi *et al.*, 2008 [104]
5-HT, gangliosides and phospholipids	Klein and Berg, 1995 [105]
Muscarinic cholinergic receptor	Tanaka *et al.*, 2003 [97]

## Review Criteria

This review included a PubMed search originally undertaken in June 2015 and updated in February 2016 for all articles published since 1950 using the main terms “chronic fatigue”, “chronic fatigue syndrome”, “myalgic encephalomyelitis” or “ME/CFS” in combination with the following terms: “aetiology”, “pathophysiology”, “antibiotics”, “probiotics”, “prebiotics”, “faecal microbiota transplantation”, “infection” “inflammation”, “immune activation”, “autoimmunity”, ”innate immunity”, “cytokines”, “toll-like receptors”, “anti-microbial peptides”, “intestinal microbiota”, ”dysbiosis” “leaky gut”, “intestinal epithelium” and “twin studies”. Studies presented in Table 1, Table 2 and Table 3 were chosen due to their large sample sizes and impact. Original papers and comprehensive reviews were emphasized as the present review covers a broad range of topics.
